# Vertebral triangle of doctor A.N. Kazantsev - double vertebral artery in V3 segment

**DOI:** 10.1016/j.radcr.2023.03.057

**Published:** 2023-04-29

**Authors:** Anton N. Kazantsev, Alexander V. Korotkikh, Maxim P. Chernyavin, Alexey P. Domke, Vasily M. Medvedev, Roman Yu. Lider, Otabek Sh. Mukhtorov, Petr D. Palagin, Alexey A. Sirotkin, Oleg V. Lebedev, Elizaveta G. Kazantsva

**Affiliations:** a Kostroma Regional Clinical Hospital named after E.I. Korolev, Kostroma, Russian Federation; b Clinic of Cardiac Surgery of the Amur State Medical Academy of the Ministry of Health of Russia, Blagoveshchensk, Russian Federation; c Clinical Hospital №1 of the Presidential Administration of the Russian Federation, Moscow, Russian Federation; d Kemerovo State Medical University, Kemerovo, Russian Federation; e Regional Dental Clinic, Kostroma, Russian Federation

**Keywords:** Third segment of the vertebral artery, Fourth segment of the vertebral artery, Duplication of the vertebral artery, Stenting of the vertebral artery, Vertebral triangle of Dr A.N. Kazantsev

## Abstract

We have described a variant of the structure of the vertebral artery. In the V3 segment, the vertebral artery bifurcated and then joined again. This building looks like a triangle. Such anatomy has not been previously described in the world literature. By the right of the first description, this anatomical formation was called the «vertebral triangle of Dr A.N. Kazantsev». This discovery was made during stenting of the V4 segment of the left vertebral artery in the most acute period of stroke.

## Introduction

There is no consensus on the need for urgent surgical interventions on brachiocephalic arteries [1], [2], [3], [4], [5]. In the presence of hemodynamically significant stenosis of the internal carotid artery (ICA), some studies come to conclusions about the safety and efficacy of both carotid endarterectomy and carotid angioplasty with stenting in the acute period (days 1-3) stroke [6], [7], [8], [9], [10]. Other authors, referring to the high risk of developing hemorrhagic transformation, in some cases call for postponing emergency brain revascularization until the acute (days 4-28) or early recovery (days 29-6 months) stroke periods [11], [12], [13], [14], [15].

This article demonstrates the successful outcome of emergency stenting of thrombosis of the V4 segment of the left vertebral artery with occlusion of the left internal carotid artery and doubling of the V3 segment of the contralateral vertebral artery (vertebral triangle of Dr A.N. Kazantsev) in the most acute period of stroke in the basin of the left middle cerebral artery.

## Clinical example

Patient G., 50 years old, male. Was admitted on an emergency basis with complaints of impaired speech, weakness in the right limbs. The real complaints appeared in the morning hours, approximately 10 hours before admission to the institution.

General condition of moderate severity. Breathing is spontaneous, vesicular, no wheezing. Hemodynamics is stable, with a tendency to hypertension. The abdomen is soft, painless on palpation.

*Neurological status*. Consciousness is clear. Cognitively reduced. Speech is an element of motor aphasia. Pupils D=S. Small-sweeping horizontal nystagmus when looking to the right. There is no paresis of the gaze. There is no hemianopia. Smoothness of the left nasolabial fold. Power paresis is not defined. No obvious sensory disturbances are presented. Muscle tone physiological, D<S. Pathological signs: Babinsky on the right. Coordinating tests: finger-nose - performs with a miss on the right, heel-knee - performs with a slight ataxia on the right. In the Romberg position - staggering, without lateralization. Sensory disturbances like gloves and socks. Meningeal symptoms are negative. The level of neurological deficit according to rating scales: Rankin - 1 point; Rivermead - 10 points; NIHSS - 5 points; Glasgow - 15 points.

**Fig. 1:**
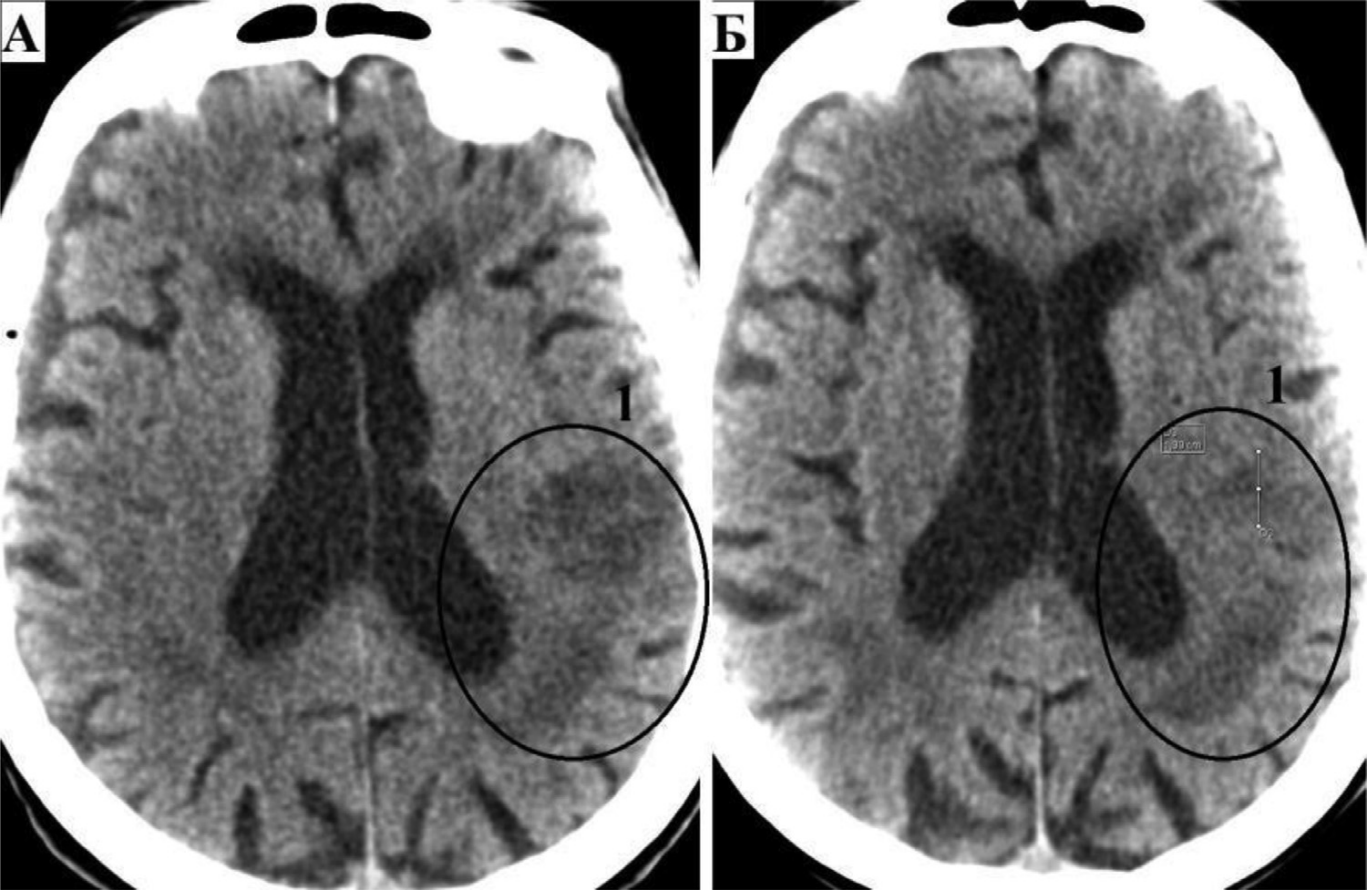
Computed tomography of the brain. (A) Computed tomography of the brain upon admission to the hospital: 1 - ischemic stroke in the basin of the left middle cerebral artery. (B) Computed tomography of the brain on day 22 after surgery: 1 - decrease in perifocal edema and the volume of the ischemic focus in the basin of the left middle cerebral artery.

According to multislice computed tomography of the brain, an ischemic stroke was detected in the basin of the left middle cerebral artery (Fig. 1).

**Fig. 2:**
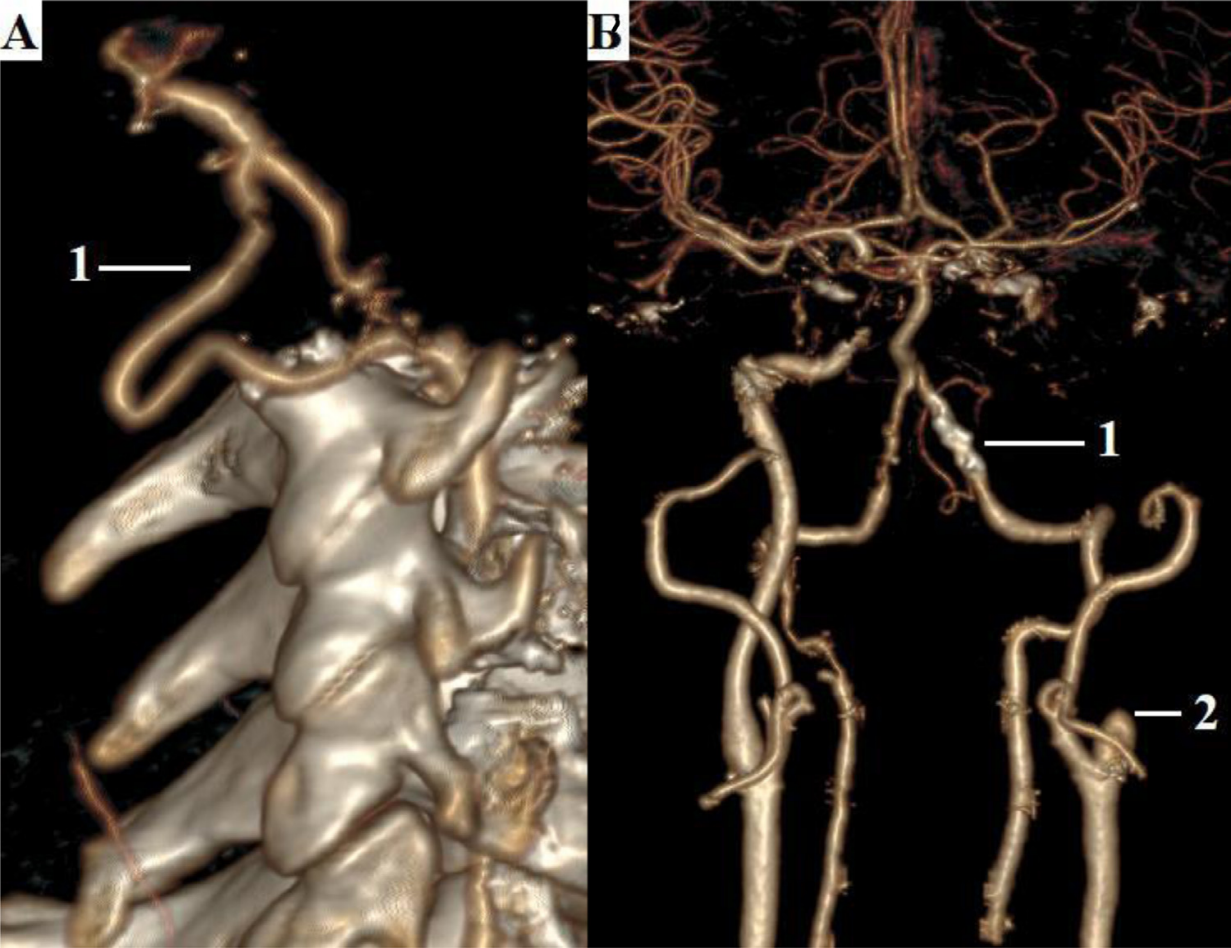
Computed tomography and angiography of extra- and intracranial arteries. (A) Lateral projection: 1 - vertebral triangle of Dr A.N. Kazantsev. (B) Direct projection: 1 - zone of thrombosis of the left vertebral artery; 2 - occlusion of the left internal carotid artery.

According to the results of color duplex scanning, occlusion of the internal carotid artery on the left was visualized. Computed tomography with angiography of extra- and intracranial arteries was performed: the left internal carotid artery was occluded throughout; thrombosis of the left vertebral artery in the V4 segment; variant structure of the right vertebral artery - doubling in the V3 segment; 50% stenosis (NASCET) of the V4 segment of the right vertebral artery; The circle of Willis is closed. In the V3 segment, the vertebral artery bifurcated and then joined again. This building looks like a triangle. Such anatomy has not been previously described in the world literature. By the right of the first description, this anatomical formation was called the "vertebral triangle of Dr A.N. Kazantsev" (Fig. 2).

A multidisciplinary council (cardiovascular surgeon, endovascular surgeon, neurosurgeon, neurologist, resuscitator, anesthetist), in view of the high risk of re-stroke, decided to perform emergency revascularization - stenting of the V4 segment of the left vertebral artery. The choice in favor of stenting was justified by a mild neurological deficit, good anatomy, and minimal size of the ischemic focus. This revascularization tactic is preferred in our medical institution. The extensive experience of such interventions (128 in 2021-2022) allows us to perform them with a low risk of any complications.

**Fig. 3:**
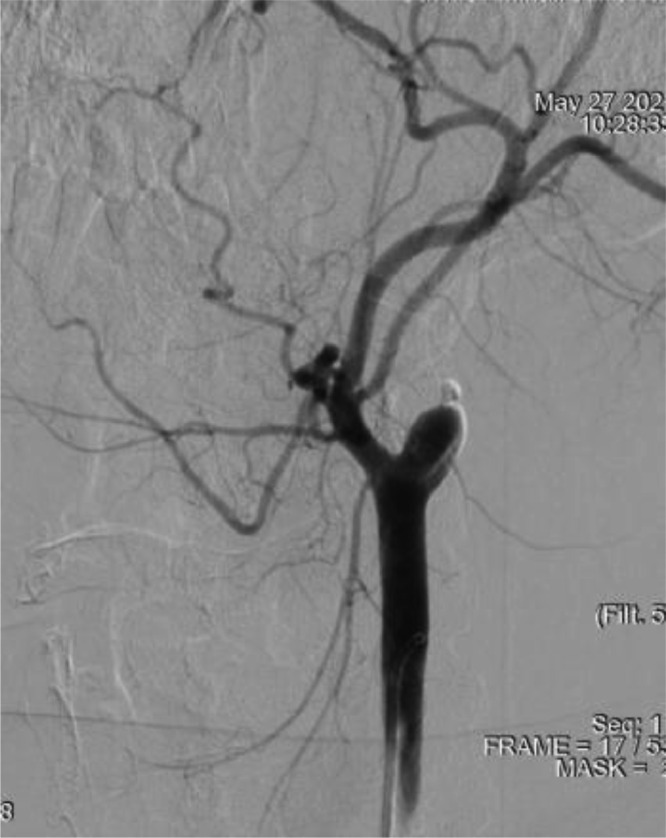
Selective catheterization of the left internal carotid artery with angiography.

Operation progress: selective angiography of the left internal carotid artery was performed: the artery was occluded (Fig. 3).

**Fig. 4:**
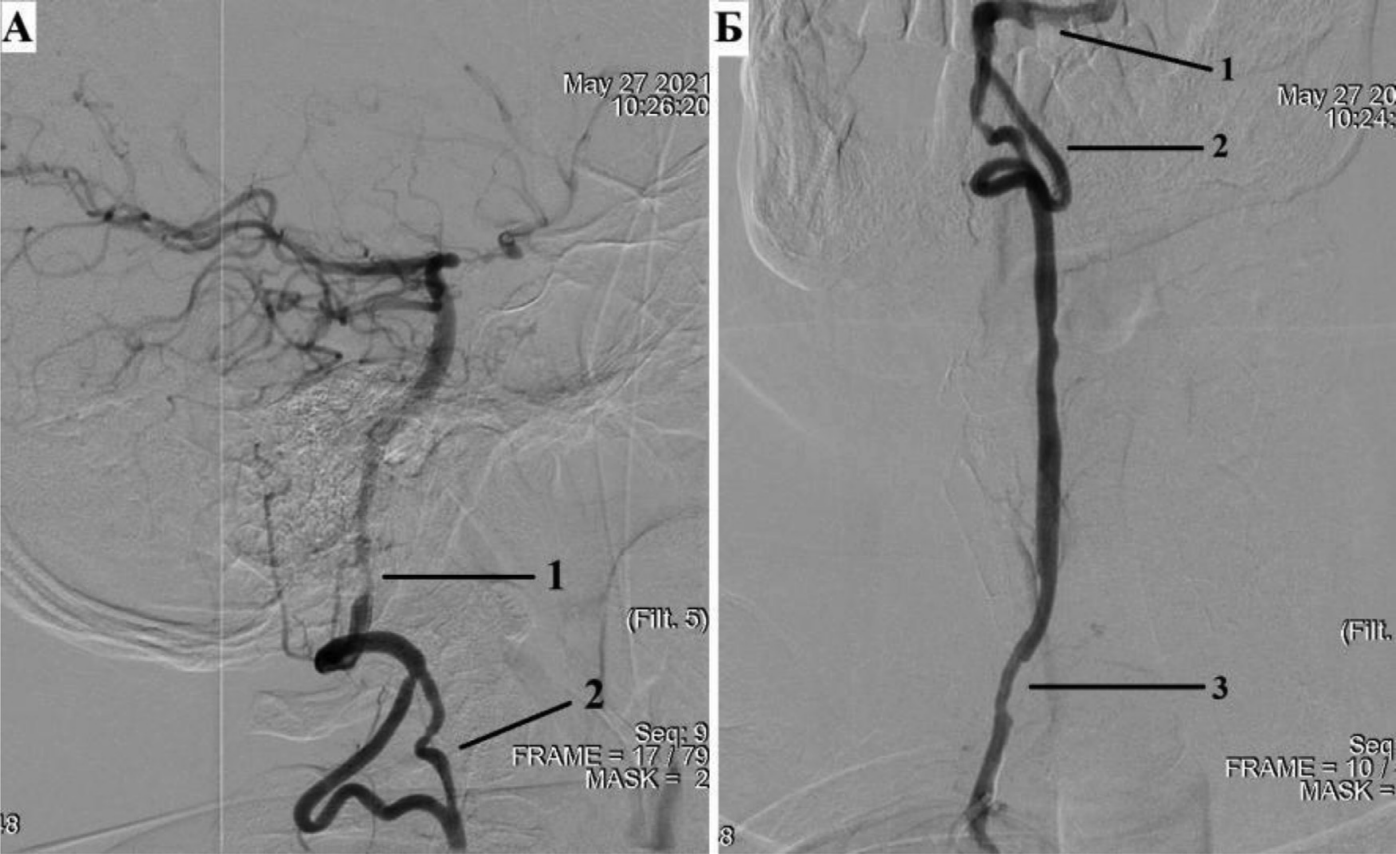
Selective catheterization of the right VA, with angiography. (A) Lateral projection; (B) Direct projection; 1 - 50% stenosis of the V4 segment of the right vertebral artery; 2 – vertebral triangle of Dr A.N. Kazantsev; 3 - 50% stenosis of the V1 segment of the right vertebral artery.

Selective angiography of the right vertebral artery was performed (Fig. 4).

**Fig. 5:**
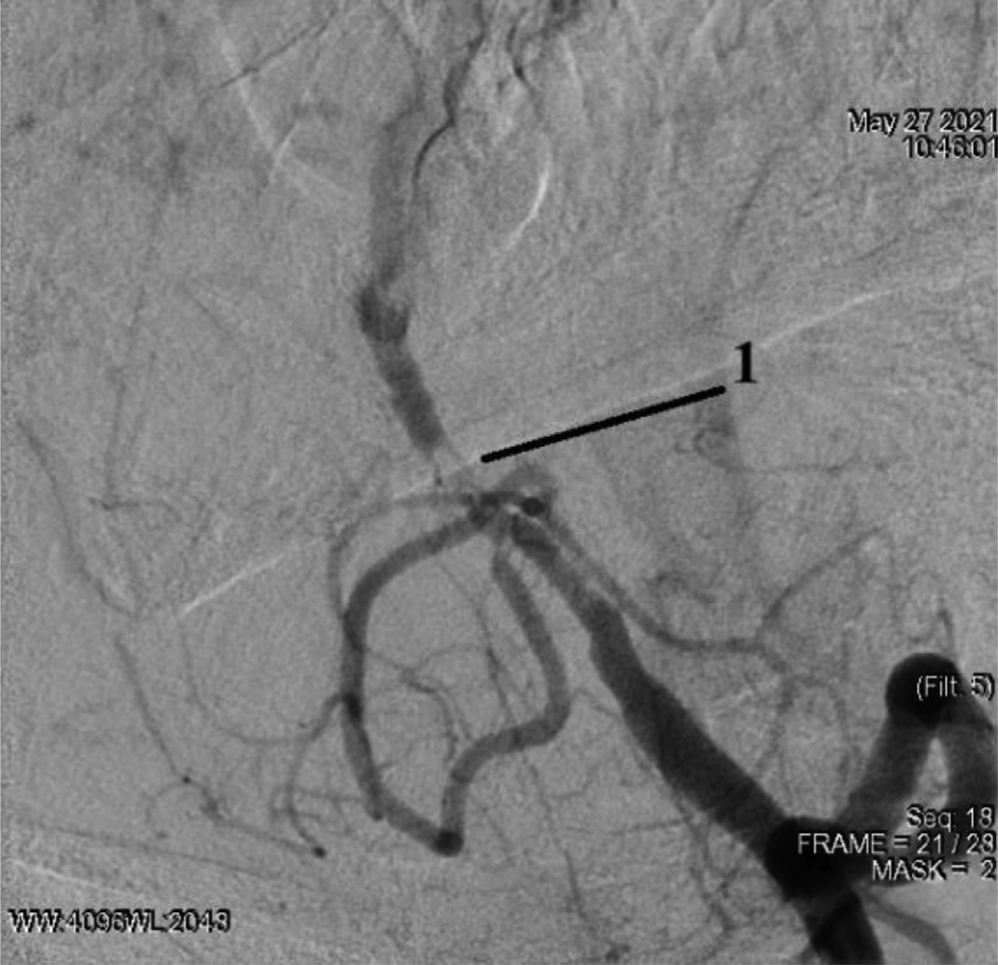
Selective catheterization of the left vertebral artery with angiography: 1 - thrombosis of the V4 segment of the left vertebral artery.

Selective angiography of the left vertebral artery was performed: thrombosis of the left vertebral artery in the V4 segment (Fig. 5).

**Fig. 6:**
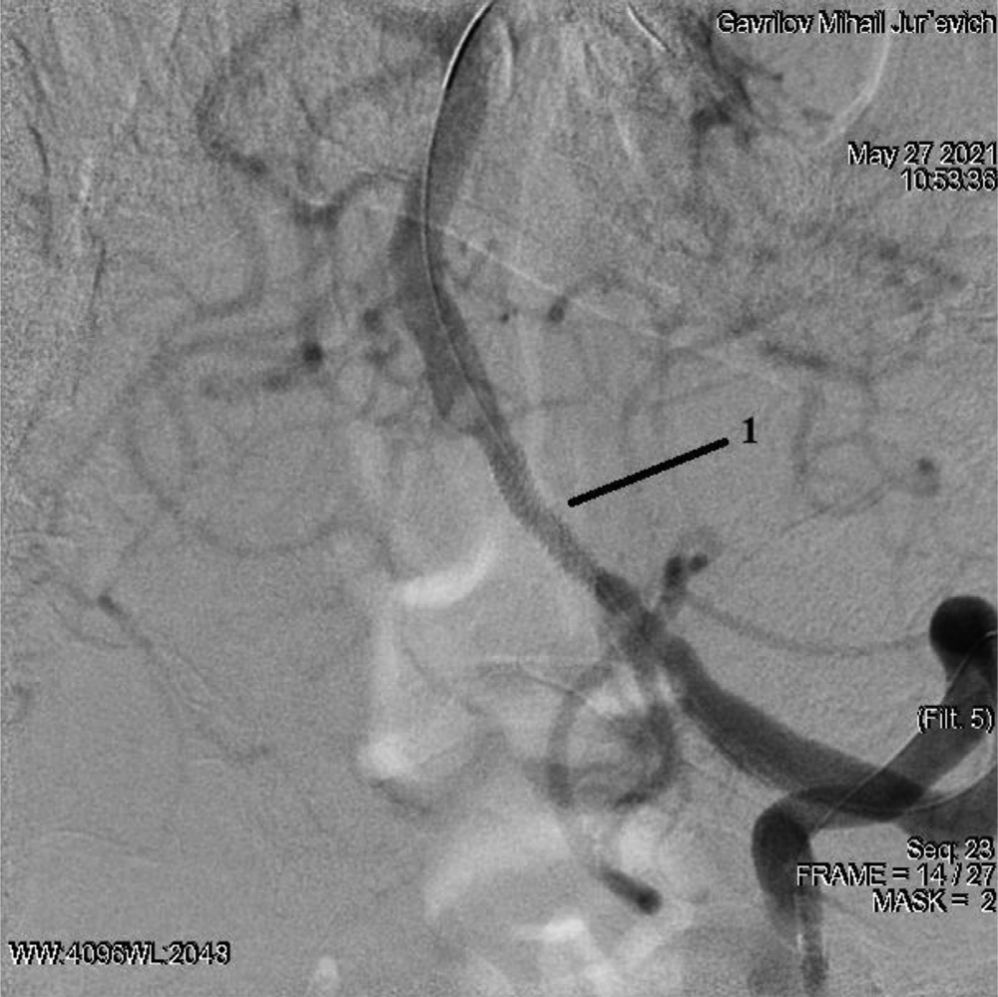
Stenting of the V4 segment of the left vertebral artery: 1 - implanted stent.

Conductor 0.014 brought into the distal parts of the main artery. According to Seldinger, a Promus Premier stent 2.5 × 12 mm (DES) was placed, positioned and deployed at a pressure of up to 12 atmospheres in the affected area of the V4 segment of the left vertebral artery (Fig. 6).

**Fig. 7:**
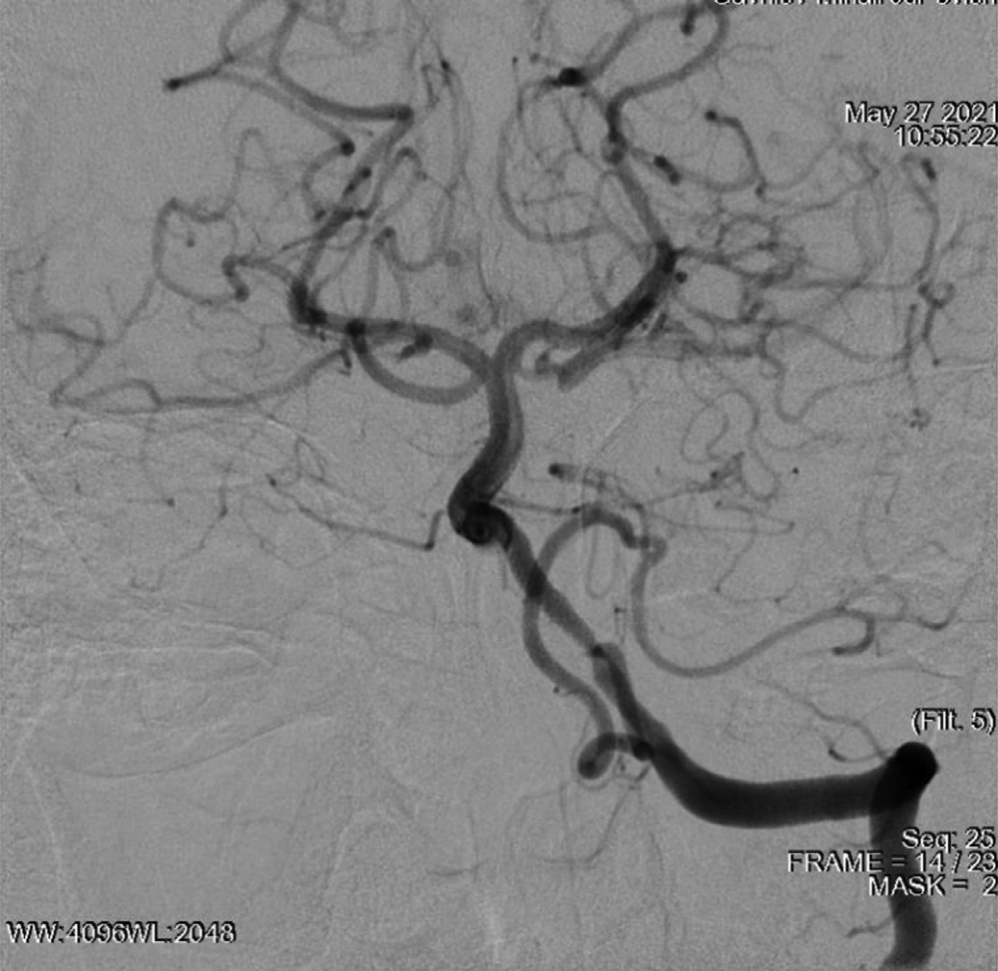
Control angiography of the intracranial arteries.

The balloon catheter has been removed. When the balloon was deflated after the stent was deployed, thrombotic masses were aspirated with a guide catheter. On the control angiography, residual stenosis of the stenting zone of the left vertebral artery was 0%, intracranial arteries showed no signs of embolism. The left carotid pool is filled through the left posterior cerebral artery (Fig. 7).

The tool has been removed. Hemostasis of the puncture site of the right common femoral artery was performed with the Angio-Seal device. Aseptic pressure bandage.

In the postoperative period, dual antiplatelet therapy was prescribed (acetylsalicylic acid 100 mg 1 time per day + clopidogrel 75 mg 1 time per day). On the 22nd day after the operation, the neurological deficit partially regressed (restoration of speech, muscle tone D = S, absence of pathological signs). Computed tomography of the brain was performed, there was a decrease in perifocal edema and the volume of the ischemic focus in the basin of the left middle cerebral artery (Fig. 1). The patient was discharged from the institution in a satisfactory condition.

## Discussion

In the framework of this work, the decision in favor of emergency stenting of the vertebral artery was due to the presence of a high risk of recurrent stroke. According to angiography, the basin of the left middle cerebral artery received blood supply mainly through the collateral branches of the circle of Willis. In the case of the development of hemodynamically significant stenosis of the internal carotid artery and with complete patency of the vertebral artery, the formation of vertebrobasilar insufficiency due to a deficiency of hemocirculation is possible [13]. In such a situation, the effect of carotid endarterectomy leads to regression of vertebrobasilar insufficiency [[16], [17], [18], [19], [20]]. In our clinical case, stenting of the left vertebral artery had a protective effect in order to eliminate circulatory insufficiency in the basin of the left middle cerebral artery. In addition, chronic occlusion of the internal carotid artery is less likely to be an inducing factor for ischemic stroke in the basin of the left middle cerebral artery. Ultimately, successful stenting of the V4 segment of the vertebral artery prevented the development of a recurrent stroke and led to a regression of neurological symptoms on day 22 after surgery.

The complexity of the intervention was that due to the pronounced tortuosity, the presence of an atherosclerotic lesion and the presence of the vertebral triangle of Dr A.N. Kazantsev, it was not possible to pass a trap through it to protect against distal embolism due to the high risk of complications (dissection, etc.). Therefore, direct stenting of the thrombosed V4 segment of the left vertebral artery was performed in combination with aspiration of thrombotic masses with a guide catheter when the balloon was deflated after the stent was deployed. This approach has become the main solution in achieving a successful outcome of emergency revascularization.

Of particular anatomical interest is the vertebral triangle of Dr A.N. Kazantsev. In all major studies on the treatment of patients with hemodynamically significant stenoses of the vertebral arteries, such topographic features have not been described [[7], [8], [9], [10],^13^]. Thus, this observation can become an additional educational element in the training of vascular surgeons.

## Conclusion

Stenting of the V4 segment of the left vertebral artery with its thrombosis in combination with aspiration of thrombotic masses with a guide catheter in the most acute period of stroke showed high efficiency and safety in a patient with vertebral triangle of Dr A.N. Kazantsev and occlusion of the left internal carotid artery. However, due to the lack of long-term results, this conclusion is relevant only for the hospital period.

## Patient consent

The patient voluntarily signed a written consent to the use of information about his treatment and personal data when writing this article.
